# Zero adjusted models with applications to analysing helminths count data

**DOI:** 10.1186/1756-0500-7-856

**Published:** 2014-11-27

**Authors:** Michael G Chipeta, Bagrey M Ngwira, Christopher Simoonga, Lawrence N Kazembe

**Affiliations:** Malawi Liverpool – Wellcome Trust Clinical Research Programme, PO Box 30096, Blantyre, Malawi; Community Health Department, University of Malawi, College of Medicine, P/Bag 360, Blantyre, Malawi; Ministry of Health, PO Box 30205, Lusaka, Zambia; Department of Statistics and Population Studies, University of Namibia, Windhoek, Namibia

**Keywords:** Excess zeros, Zero–inflated, Two–part model, Count data, Statistical methods, Hurdle, Negative binomial

## Abstract

**Background:**

It is common in public health and epidemiology that the outcome of interest is counts of events occurrence. Analysing these data using classical linear models is mostly inappropriate, even after transformation of outcome variables due to overdispersion. Zero-adjusted mixture count models such as zero-inflated and hurdle count models are applied to count data when over-dispersion and excess zeros exist. Main objective of the current paper is to apply such models to analyse risk factors associated with human helminths (*S. haematobium*) particularly in a case where there’s a high proportion of zero counts.

**Methods:**

The data were collected during a community-based randomised control trial assessing the impact of mass drug administration (MDA) with praziquantel in Malawi, and a school-based cross sectional epidemiology survey in Zambia. Count data models including traditional (Poisson and negative binomial) models, zero modified models (zero inflated Poisson and zero inflated negative binomial) and hurdle models (Poisson logit hurdle and negative binomial logit hurdle) were fitted and compared.

**Results:**

Using Akaike information criteria (AIC), the negative binomial logit hurdle (NBLH) and zero inflated negative binomial (ZINB) showed best performance in both datasets. With regards to zero count capturing, these models performed better than other models.

**Conclusion:**

This paper showed that zero modified NBLH and ZINB models are more appropriate methods for the analysis of data with excess zeros. The choice between the hurdle and zero-inflated models should be based on the aim and endpoints of the study.

## Background

It is common in public health and epidemiology that the outcome of interest is counts of occurrence of events. In human helminth disease research, such as hookworms or *Schistosoma haematobium* studies, the outcome of interest includes number of egg counts in a urine or stool of a human sample. Such count data are typically very skewed and exhibit many zero count observations [1]. It is well known that analysing these data using classical linear models is mostly inappropriate, even after transformation of outcome variables [1–3].

The natural choice for count data analysis is the Poisson regression [4, 5]. However, the underlying Poisson distribution has several limitations that are often neglected, including lack of allowance for over-dispersion [4, 5]. One frequent manifestation of lack of allowance for over-dispersion is that the incidence of zero counts is greater than expected for the Poisson distribution. The excess zeros can occur as a result of clustering. Therefore, it is worthwhile to consider the mechanism by which over-dispersion occurs and use more flexible models such as the heterogeneous Poisson models and zero-modified models. The most commonly used heterogeneous Poisson distribution is the negative binomial [2, 6, 7] which loosens Poisson restrictions by allowing the expected number of events to be a function of some unobserved random variable that follows a gamma distribution [8]. Alternatively, to overcome this violation we extend the Poisson model to have an over-dispersion parameter, through a hierarchical model that introduces a gamma prior on the mean parameter [5].

One could also use zero-adjusted mixture models such as zero-inflated (ZI) and hurdle count models [8, 9] which are applied to count data when over-dispersion exists and excess zeros are indicated [5, 6, 10, 11]. ZI models include zero inflated Poisson (ZIP) [12] and zero inflated negative binomial (ZINB), whereas hurdle models include Poisson logit hurdle (PLH) and negative binomial logit hurdle (NBLH) [5]. The PLH is also known as zero altered Poisson (ZAP). The PLH and NBLH can be considered as mixture models in which the complete distribution of the outcome is represented by two separate components, a first part modelling the probability of excess zeros and a second part accounting for the non-excess zeros and non-zero counts [13], thus estimating two equations. In contrast to ZI models, the zero and non-zero counts are separated in hurdle models. More specifically, in both zero-modified models, a logit model with binomial assumption is used to determine which of the two processes generates an observation [2]. A further extension to hurdle models is developed in [14], in which the processes generating zeros and positives are not constrained to be the same, and that hurdles may not be restrictively be at zero. The NB regression models have been widely used to analyse human helminths infection intensity data [15, 16], but recently, there is more and more research that is analysing human helminth data using zero-modified models highlighted above. Be that as it may, these models offer a natural epidemiological interpretation of disease infectivity and transmission intensity as we expand below. For instance, one may assume that a proportion of individuals have no chance of being infected, as they are not exposed. In other words, there is a process which determines whether an individual is likely to be infected at all (infection probability) and a second process determining the number of excreted eggs among those who are at risk of infection (infection intensity) [17]. ZIP models assume that number of excreted eggs follows a Poisson distribution. ZINB models assume that the number of eggs among those who are at risk of infection has a negative binomial distribution [11]. The hurdle model has an interpretation as a multiple-part model, the simplest being the two-part model; the first part as binary outcome model and the second part as a truncated count model. Such a partition permits the interpretation that positive observations arise from crossing a zero hurdle or zero threshold. In principle, the threshold need not be at zero; it could be any value. Further, it need not be treated as known. The zero value has special appeal, however, because in many situations it partitions the population into sub-populations in a meaningful way [13]. In contrast to the ZI model, zero and non-zero counts are separated into hurdle models [1], a fact that makes them very useful in epidemiological studies.

The main objective of the research reported in this article is to apply zero-adjusted mixture count data models to analyse risk factors associated with human helminths (*S. haematobium*) particularly in a case where there is a high proportion of zero counts. Two actual data sets, one from Malawi and another from Zambia, with different levels of zero counts were used in the current analysis, and they corresponded to eggs (counts) excreted by an individual. Poisson, negative binomial, and two zero modified (ZI and hurdle) parameterisations for the Poisson and negative binomial distributions were fitted to both data sets and results were compared.

## Methods

### Count data models

Various statistical models have been developed to model *S. haematobium* disease burden. These are Poisson, negative binomial (NB), ZI (Poisson and negative binomial), logit hurdle (Poisson and negative binomial). Below, a brief outline for each of the models mentioned above is given.

#### Poisson model

Poisson regression is traditionally conceived as the basic count model upon which a variety of other count models are based [5, 18]. Poisson distribution is characterized as:
1

where random variable *k* is the count response and parameter *λ* is the mean. Unlike most other distributions, Poisson does not have a distinct scale parameter.

The standard Poisson distribution, which assumes equal variance and mean, is not appropriate to fit the observed egg counts since variance of the counts is much larger than their mean. Violations of equidispersion indicate correlation in the data, which affects standard errors of the parameter estimates. Model fit is also affected. When such a situation arises, modifications are made to the Poisson model to account for discrepancies in the goodness of fit of the underlying distribution. Negative binomial (NB) is normally used to model overdispersed Poisson data. A dispersion parameter is included in the NB model to cater for overdispersion by allowing the variance to be greater than the mean and accommodate the unobserved heterogeneity in the count data.

Poisson regression model derives from Poisson distribution and relates *λ*, *β*, and *x* through:
2

Here, *xβ* is the linear predictor, which is also symbolized as *η* within the context of generalized linear models (GLM).

#### Negative binomial model

The NB model is employed as a distribution form that relaxes the equidispersion restriction of the Poisson model [19]. The NB distribution is given by:
3

where random variable *Y* has a NB distribution with parameters *τ* ≥ 0 and *λ* ≥ 0. Its mean and variance are given by:
4

and
5

Since *λ* ≥ 0, the variance of NB distribution generally exceeds its mean ("over-dispersion") [20]. It has since been proposed to model excessive variation in helminth egg counts [21]. NB regression models have been widely used to analyse helminths infection intensity data [7, 15, 16]. However, distributional problems affect both models (Poisson and NB) such as over-dispersion resulting from specification errors in the systematic part of the regression model, hence NB models themselves may be overdispersed [5]. Nevertheless, both models can be extended to accommodate any extra correlation or dispersion in the data that result in a violation of distributional properties of each respective distribution. The enhanced Poisson or NB model can be regarded as a solution to a violation of distributional assumptions of the primary model (1). For a better fit, an overdispersed model that incorporates excess zeros should serve as an alternative [6]. Zero modified models such as ZI and hurdle count models are capable of incorporating excess zeros. They are applied to count data when over-dispersion exists and excess zeros are indicated [5, 10].

#### Zero inflated poisson

In ZIP regression, the counts *Y*_*i*_ equal 0 with probability *p*_*i*_ and follow a Poisson distribution with mean *λ*_*i*_, with probability 1 - *p*_*i*_ where *i* = 0,1,2,…,n. ZIP model can thus be seen as a mixture of two component distributions, a zero part and no-zero components, given by:
67

From Equation (6), zero observations arise from both zero-component distribution and Poisson distribution. The zero-component distribution is therefore related to modeling ‘excess’ or ‘inflated’ zeros that are observed in addition to zeros that are expected to be observed under the assumed Poisson distribution. To assess impact of covariates on the count distribution in a ZIP model, *p*_*i*_ and *λ*_*i*_ can be explicitly expressed as a function of covariates. The most natural choice to model probability of excess zeros is to use a logistic regression model:
8

where *x*_*i*_ represents a vector of covariates and *β* a vector of parameters. The effect of covariates on count data excluding excess zeros can be modeled through Poisson regression:
9

Mean and variance of ZIP model are:
1011

#### Zero inflated negative binomial

The ZINB distribution is a mixture distribution assigning a mass of *p* to ‘extra’ zeros and a mass of (1 - *p*) to a negative binomial distribution, where 0 ≤ *p* ≤ 1. The ZINB distribution is given by:
12

The mean and variance of the ZINB distribution are:
1314

Observe that this distribution approaches the zero inflated Poisson distribution and the negative binomial distribution as *τ* → *∞* and *p* → 0, respectively. If both  and *p* ≈ 0 then the ZINB distribution reduces to the Poisson distribution. The ZINB regression model relates *p* and *λ* to covariates, that is,
15

and
16

where *i* = 1,2,…,*n* and *x*_*i*_ and *z*_*i*_ are d- and q-dimensional vectors of covariates pertaining to the *i*th subject, and with *β* and *γ* the corresponding vectors of regression coefficients, respectively.

#### Poisson logit hurdle

PLH model is a two-component model comprising of a hurdle component models zero versus non-zero counts, and a truncated Poisson count component is employed for the non-zero counts:
1718

*p*_*i*_ models all zeros. For PLH model, the most natural choice to model probability of zeros is to use a logistic regression model:
19

while the effect of covariates *z*_*i*_ on strictly positive (that is, censored) count data are modeled through Poisson regression:
20

#### Negative binomial logit hurdle

Similarly, for the hurdle models, the NBLH can be used instead of Poisson distribution above in case of over-dispersion:
2122

where k = 1, 2, 3,...

The most natural choice to model probability of excess zeros is to use a logistic regression model:
23

Impact of covariates on count data modeled through NB regression:
24

Given *π* = *Pr*(*Y* > 0), the probability of a nonzero response with *η* as given in the follow up to Equation 2, the expected value and the corresponding variance are given by:
2526

While both ZI and hurdle models need distributional assumptions for their count component, both classes differ with respect to their dependencies of estimation of parameters of "zero" component on these assumptions [1]. Unlike ZI model, estimation of parameters *β* related to *p*_*i*_ in the hurdle model is not dependent on estimation of parameters *γ* related to *λ*_*i*_. Hence, if assumptions about the (truncated) Poisson/negative binomial model are violated (for example due to extreme outlying observations), the hurdle model will, in contrast to ZI model, still yield consistent estimators for parameters in the logit part of the model (if correctly specified) [1]. Much as the hurdle model will be consistent in the absence of a good model for the non-zero counts, however, one of its weaknesses is that it assumes all zeros to come from a single population.

### Model comparison

For comparison of non-nested models based on maximum likelihood, to choose the best fitting model, Akaike’s information criterion (AIC) has been proposed for model selection criteria based on the fitted log-likelihood function [13, 22, 23]. As a measure of the relative goodness of fit of a statistical model, AIC not only rewards goodness of fit, but also includes a penalty that is an increasing function of the number of estimated parameters. Since the log-likelihood is expected to increase as parameters are added to a model, the AIC criteria penalize models with larger *q*. This penalty function may also be a function of *n*, the number of observations [13]. This penalty discourages over-fitting. The AIC is specified as:
27

where *L* is the maximized value of the likelihood function for the estimated model, with *q* being equal to number of degrees of freedom used in the model and 2 is a tuning parameter meant to balance the information in the model based on the degrees of freedom with information in the residuals. A model with lowest AIC is preferred [22]. Several alternatives of AIC also exist, viz Bayesian information criteria (BIC) and Consistent Akaike’s information criterion (CAIC) [13]. AIC is optimal in selecting the model with the least mean squared error while BIC is not asymptotically optimal [24]. An AIC, CAIC or BIC difference of less than 4 indicates that the two competing models are indistinguishable, while a value difference of 4 to 10 suggests moderate superiority of one model against the other, and an AIC, CAIC or BIC difference of greater than 10 implies that for two competing models, one model is better than the other [23].

### Applications

#### Case data

Two datasets were used as a case study in the analysis of count data on human helminths. The first data set came from a study carried out in 2004 in Chikwawa district, in the Lower Shire Valley-southern Malawi. The Chikwawa data were collected from a rural population mainly engaged in subsistence farming. The area lies between 100 and 300m above sea level. The rainy season extends from December to March. Temperatures can rise up to 50°C in months preceding rainy season. The design and methods for data collection are described in [25, 26].

Briefly, data were collected in eighteen (18) villages, purposively selected from the control and intervention arms of a cluster randomized study design. There was only one round of treatment following community based and house to house approaches for mass drug administration (MDA). Over 90 percent of the eligible population were treated. All infected participants in non-intervention arm received appropriate treatment. After the follow-up assessment, both arms had mass treatment. In the study, polyparasitism was considered basing on the number of species an individual was hosting. The focus was on *S.haematobium*, *S.mansoni*, hookworm and *Ascaris*. Polyparasitism is the epidemiology of multiple species parasite infections [26]. Ten percent of the households were randomly selected from the villages for baseline survey using random number tables [25].

Subjects for geo-helminth survey were selected using a two stage-design. In short, at first stage villages were selected, then at second stage, sample of households was listed and chosen. In the selected households, all members aged one year and above were invited to participate. Consenting individuals had their demographic details completed and were given full body clinical examinations (except genitals for females) for chronic manifestations of human helminths. In addition they had anthropometric measurements taken and were asked to provide a single fresh stool and urine sample. All individuals (aged >1 year) were requested to provide a finger prick blood sample [21]. All body samples were analyzed in the laboratory using Katz test to determine geohelminth infection and intensity. Further details are provided in [25].

The second data, was school-based, collected in a cross-sectional study that was carried out in Kafue and Luangwa Districts of Lusaka province, Zambia in 2004. The two districts were selected on the basis of their ecological representativeness of the country in general [27]. In each of these districts, ten (10) primary schools were selected. Approximately 100 schoolchildren, aged 6 to 15 years, were recruited from every school. The altitude and geographical location (longitude, latitude) of the surveyed schools were obtained from the archives of the Zambian Survey Department (2003). Further details of the study design are given elsewhere in [27].

Data on *S.haematobium* prevalence and intensity were obtained using a Quantitative Filtration technique [28] to process duplicate urine samples collected about mid-morning. Two laboratory technicians prepared and read specimen filters. Both technicians read each specimen independently. This was useful in increasing sensitivity of the technique, particularly where egg intensity was low. All pupils found infected were treated with praziquantel (40 mg/kg body weight). Individual data sheets were used to collect ancillary information on each child examined.

In addition, data on intermediate host snails were also obtained through field collections and laboratory-based species identification. Sampling of potential schistosomiasis transmission sites was done based on water body proximity to respective primary school, that is, the nearest likely infection source. These water points were also qualified by relevant local people as the most frequented water contact points for both domestic and/or livestock [27].

Statistical fitting for all models was carried out using Political Science Computational Laboratory (PSCL) package [29] in R statistical software (The R Foundation for Statistical Computing, Version 2.14.0).

### Ethical approval

The study that collected data from Chikwawa, Malawi received ethical clearance from Malawi’s College of Medicine Research Ethics Committee (COMREC) [25]. Individual informed consent was orally obtained from each participant or (if they were aged <16) from one of their parents or a legal guardian. COMREC approved oral informed consent because the study was determined to be of minimal risk. The consent process was a four stage process. First stage, oral informed consent was obtained at the traditional authority (TA) level. Second stage, at village head level and third stage at the household level from the head of the household and fourth at individual level from each individual in the household (if applicable) else from parent/guardian if an individual was aged <16. Registers were kept for documentation whereby, for each individual in the selected household, a column was kept to indicate whether an individual had orally consented to participate in the study or not. Similarly, in the Zambia study, ethical approval for data collection on urinary Schistosomiasis in school children was received from University of Zambia Ethics Committee [27].

## Results

### *S. haematobium*in Chikhwawa District, Malawi

Table 1 gives summary statistics for study participants who had outcomes of interest. The study had 1642 participants of which 55.4% were female. The mean age (years) of 32.4 (standard deviation: 22.8). Of these, 324 had hookworm representing 19.7% of sample population, 71 of these had *S. mansoni* representing 4.3% and 233 had *S. haematobium* representing a prevalence of 14.2%.

**Table 1:** **Characteristics for** ***S. Haematobium*** **participants (N = 1642) in Chikwawa, Malawi**

Variable	Mean (Std. Dev)		Number (%)
*Outcome*			
S. Haematobium			233 (19.35)
S. Mansoni			71 (5.02)
Hookworm			324 (22.9)
*Age (years)*	32.36 (22.79)		
*Sex*			
Female			909 (55.36)
Male			733 (44.64)
*Education level*			
None			745 (45.37)
Primary			850 (51.77)
Secondary			47 (2.86)
*Village type*			
Intervention			831 (50.61)
Control			811 (49.39)
*Fishing*			
Yes			1,421 (86.54)
No			221 (13.46)
*Garden*			
Yes			960 (58.47)
No			682 (41.53)
*Occupation*			
Farmer			733 (44.64)
Other			909 (55.36)
*Polyparasitism*			
None			807 (49.15)
One			594 (36.18)
Two			200 (12.18)
Three			38 (2.31)
Four			3 (0.18)

**Figure 1:**
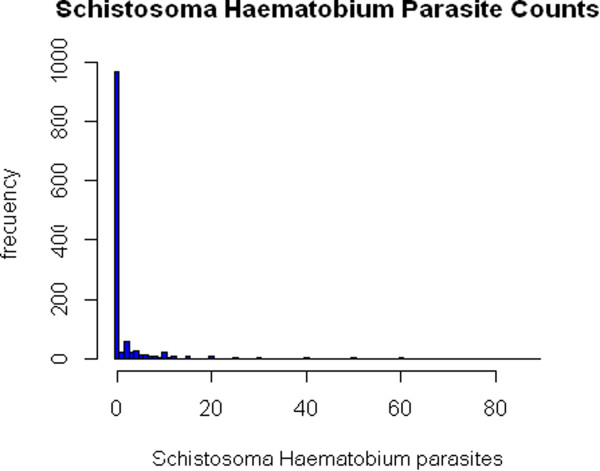
**Distribution of egg counts for** ***S. haematobium*** **in Malawi study.**

Figure 1 shows that a large proportion of individuals i.e. 85.8% for *S. haematobium* were "zero egg excretors". The likelihood ratio test for over-dispersion between Poisson and Negative binomial at *α* = 0.05 showed a critical value test statistic = 2.7 with a *χ*^2^ test statistic = 10606.5, p-value < 0.001. Indeed, there was overwhelming evidence of overdispersion. This was confirmed by the presence of excess zeros (Figure 1).

#### Model comparison

Using the AIC and zero capturing, the predicted counts using the ZINB and NBLH indicate a closer fit with the observed values. In Table 2, AIC results show that the ZINB and NBLH are similar and offer a better fit compared to using Poisson Logit Hurdle (PLH) or a negative binomial (AIC = 3,482 for NBLH; AIC = 3, 484 for ZINB whereas AIC = 6,854 for PLH and AIC = 3,576 for NB respectively). With regards to zero capturing, the Poisson model was again not appropriate as it could only capture 515 of the zeros whereas the zero adjusted based models were much better in capturing the zero counts. The following models performed relatively similar: Negative Binomial, ZIP, ZINB, PLH with NBLH model capturing 971 zeros which were equal to the observed (Table 3). From these results, NB modified based models (NBLH and ZINB) offered the best fit to zero inflated helminth data in terms of the AIC (minimum value for all the models fitted) and are indistinguishable with respect to fit, AIC difference of less than 4 between the two (see Table 2). NBLH was used for fitting a final model to model helminth infection intensity and determination of factors that foster infections due to its ease of implementation and its direct link with the observed data. Besides, NBLH has an advantage over ZINB as it has the lowest AIC despite the difference of less than 4.

**Table 2:** **Model comparison using Akaike Information Criterion (AIC) for Malawi study data**

	Poisson	Neg. Bin.	ZIP	ZINB	PLH	NBLH
**AIC**	14,182	3, 576	6, 854	3, 484	6, 854	3, 482

**Table 3:** **Zero count capturing in the Malawi study model**

Observed	Poisson	Neg. Bin	ZIP	ZINB	PLH	NBLH
971	515	968	970	969	970	971

#### Modelling and interpreting main effects

**Fixed effects: infection probability** Table 4 provides estimates for the fixed effects from the hurdle model, with significant factors at 5% significance level given in bold type. The probability of infection was found to be associated with age (Odds Ratio [OR] = 0.97, 95% Confidence Interval [CI]: 0.96–0.99), the risk of infection decreased with age. The risk of infection was low in males than in females (OR = 0.61, 95% CI: 0.41–0.89). Infection probability was also found to be associated with village type; whether one was in the intervention area or control area (OR = 0.38, 95% CI: 0.26–0.54). Those in the intervention area were at a reduced chance of infection relative to those in control area. We also noted that chances of infection were increasing with number of parasites an individual was hosting (OR = 7.30, 95% CI: 5.56–9.59).

**Table 4:** **Fixed effects estimates for NBLH model for** ***S. Haematobium*** **in Malawi study**

	Infection probability odds ratio (OR)	95% CI	Infection intensity relative risk (RR)	95% CI
*Intercept*	0.13	(0.06, 0.29)	11.72	(5.70, 24.08)
Age	0.97	(0.96, 0.99)	0.96	(0.95, 0.98)
*Sex:*				
Female	1.00		1.00	
Male	0.61	(0.41, 0.89)	1.03	(0.72, 1.47)
*Education:*				
None	1.00		1.00	
Primary	1.18	(0.81, 1.71)	1.54	(1.08, 2.19)
Secondary	1.37	(0.41, 4.60)	0.34	(0.11, 1.06)
*Village Type:*				
Control	1.00		1.00	
Intervention	0.38	(0.26, 0.54)	0.81	(0.58, 1.13)
*Fishing:*				
No	1.00		1.00	
Yes	0.73	(0.44, 1.20)	0.68	(0.45, 1.03)
*Garden:*				
No	1.00		1.00	
Yes	1.34	(0.90, 1.99)	1.21	(0.82, 1.81)
*Occupation:*				
Other	1.00		1.00	
Farmer	0.61	(0.35, 1.06)	1.83	(1.16, 2.91)
Polyparasitism	7.30	(5.56, 9.59)	0.87	(0.70, 1.08)

**Fixed effects: Infection intensity** From Table 4, it was observed that infection intensity reduced with an increase in age (Relative Risk (RR) = 0.96, 95% CI: 0.95–0.98). There was no difference in terms of infection intensity between males and females (RR = 1.03, 95% CI: 0.72–1.47). Primary school children showed a high infection intensity relative to those that are in pre-school level (RR = 1.54, 95% CI: 1.08–2.19) whereas those in secondary level showed a reduced infection intensity (RR = 0.34, 95% CI: 0.11–1.06) but not significant at 5% level. A positive association was also observed between those who did fishing in Shire river relative to those who did not fish (Table 4). We observed an increased risk of infection intensity farmers compared to non-farmers (RR = 1.83, 9% CI: 1.16–2.91).

### *S. haematobium*in school children in Lusaka Province, Zambia

Table 5 gives characteristics of study population. A total of 2040 school children aged 6 to 15 years were enrolled into the study from 20 selected primary schools in the two districts, Kafue and Luangwa, 1909 (93.5%) provided urine samples for parasitological examination. The remaining children 131 (6.5%) did not provide urine samples for examination. Overall *S. haematobium* prevalence rate for two districts was 9.6% (range: 0 – 36.1). Infection intensity had a mean of 31.4 eggs/10 ml (range: 0 – 120 eggs/10 ml). However there was a significant difference in the mean intensity of infection, with 40.2 (range: 3 – 53.1 eggs/10 ml) observed in Kafue district and 22.6 (range: 0 – 116.0 eggs/10 ml) in Luangwa district. For Schistosomiasis, a large proportion of individuals were "non egg excretors" (84.6%).

**Table 5:** **Characteristics and intensity of infection with** ***S. haematobium*** **in 2040 children from 20 schools in Lusaka Province, Zambia, 2004**

Variable	Mean (std. dev)	Number (%)
*Intensity of infection*		
No infection (0 eggs/ml: epm)		1726 (84.6)
Light infection (1-100 epm)		139 (6.8)
Mod/heavy infection (> 100 epm)		44 (2.2)
*Age (years)*	9.98 (2.14)	
6-9 years		1130 (55.4)
10-15 years		900 (44.1)
*Sex*		
Female		1027 (50.4)
Male		1000 (49.0)
*Altitude*		
Plateau		723 (35.4)
Valley		1316 (64.5)
*NDVI*	138.2 (5.1)	
*TMAX*	19.6 (2.9)	
*Snail abundance (B. globosus)*	25.3 (29.9)	

Similar to results from Malawi study, gender showed to be insensitive to urinary Schistosomiasis prevalence and intensity with a *χ*^2^ = 2.4 and a p-value = 0.124. Age showed marginal differences between the 6-9 years and 10-15 years age groups, *χ*^2^ = 4.0 with p-value = 0.059. With a *χ*^2^ =29.5, altitude showed significant difference between valleys and plateaus in influencing infection prevalence and intensity. Normalised difference vegetation index (NDVI) (t = 1280.47, p-value < 0.001) showed a significant impact on urinary Schistosomiasis. Again, Tmax (t = 282.26) and snail abundance (t = 30.82) both with p-values < 0.001 also showed significant impact on urinary Schistosomiasis.

#### Model comparison

Using the AIC and zero capturing, AIC results show that the NBLH and ZINB offer a better fit compared to using Poisson Logit Hurdle (PLH) or a negative binomial (AIC = 3,230 for NBLH and 3,232 for ZINB; whereas AIC = 14,8734 for PLH and AIC = 3,250 for NB respectively), see Table 6. With regards to zero capturing, the Poisson model was again not appropriate as it could only capture 183 of the zeros whereas the zero adjusted models were much better in capturing the zero counts. The NBLH, ZINB, PLH and ZIP models captured 1705 zeros which were equal to the observed (Table 7). Since NB modified based models (NBLH and ZINB) offered the best fit to zero inflated helminth data in terms of the AIC (minimum value for all the models fitted), NBLH therefore was used for fitting a final model to model helminth infection intensity and determination of factors that foster infections.

**Table 6:** **Model comparison using Akaike Information Criterion (AIC) for Zambia study data**

	Poisson	Neg. Bin.	ZIP	ZINB	PLH	NBLH
**AIC**	351,690	3,250	148,734	3,232	148, 734	3,230

**Table 7:** **Zero count capturing in Zambia study model**

Observed	Poisson	Neg. Bin	ZIP	ZINB	PLH	NBLH
1705	183	1704	1705	1705	1705	1705

#### Modelling and interpreting main effects

**Fixed effects: Infection probability** From Table 8, significant factors at 5% significance level are given in bold type. The probability of urinary Schistosomiasis infection was shown to have a significant association with age (OR = 0.69, 95% CI: 0.50–0.94) with lower risk in younger children. Infection probability showed a positive association with sex (OR = 1.17, 95% CI: 0.86–1.60) though not significantly different between females and males. We observed a negative association between infection probability and altitude (OR = 0.37, 95% CI: 0.25–0.53) with those in the valley at an increased risk of infection. Maximum temperature (TMAX) showed an association with probability of infection though not significant at 5% level (OR = 0.99, 95% CI: 0.94–1.04). We also observed a positive relationship between snail abundance and risk of infection, (OR = 1.01, 95% CI: 1.00–1.01). Marginal positive association was observed between urinary Schistosomiasis and NDVI (the mean Dec–Nov biannual composites of NDVI) (OR = 1.04, 95% CI: 1.00–1.07).

**Table 8:** **Probability and intensity of infection with** ***S. haematobium*** **in 2040 children from 20 schools in Lusaka Province, Zambia, 2004**

	Infection probability odds ratio (OR)	95% CI	Infection intensity relative risk (RR)	95% CI
*Intercept*	0.01	(0.00, 0.65)	13.03	
*Age:*				
10-15 years	1.00		1.00	
6-9 years	0.69	(0.50, 0.94)	0.55	(0.25, 1.19)
*Gender:*				
Female	1.00		1.00	
Male	1.17	(0.86, 1.60)	1.28	(0.57, 2.87)
*Altitude:*				
Plateau	1.00		1.00	
Valley	0.37	(0.25, 0.53)	0.11	(0.04, 0.28)
*TMAX*	0.99	(0.94, 1.04)	0.84	(0.75, 0.94)
*NDVI*	1.04	(1.00, 1.07)	1.07	(0.99, 1.16)
*Snail abundance*	1.01	(1.00, 1.01)	1.00	(0.99, 1.01)

**Fixed effects: infection intensity** From Table 8, we observed that infection intensity was marginally associated with age (RR = 0.55, 95% CI: 0.25–1.19). It was observed that temperature was negatively associated with urinary Schistosomiasis intensity (RR = 0.75, 95% CI: 0.75–0.94), as well as altitude (RR = 0.11, 95% CI: 0.04–0.28). Snail abundance was marginally associated with infection intensity (RR = 1.00, 95% CI: 0.99–1.01). We also observed a positive association between infection intensity and NDVI (mean Dec-Nov biannual composites of NDVI) (RR = 1.07, 95% CI: 0.99–1.16) as well as gender (RR = 1.28, 95% CI: 0.57–2.87) albeit both not significant at 5% level.

## Discussion

This paper considered several count data models to quantify factors associated with prevalence and intensity of *S. haematobium* infection using two different datasets inflated with zeros. Factors considered include such as age, sex, education level, village type, fishing in rivers, working in gardens, occupation and polyparasitism. All the models considered modelled intensity by using the actual egg counts as the response. An alternative approach to modelling intensity of infection is derived by categorizing egg-counts into groups of no infection, light and moderate/high infection based on egg counts in the urine samples. The competing models were compared to each other in terms of AIC, estimated regression coefficients and zero count capturing.

Results from model comparison and selection showed that NB modified based models (NBLH and ZINB) are better for modeling excess zeros as they competed well in terms of both AIC as well as zero count capturing. Our results illustrate, for the observed data, that the ZINB and NBH models are preferred but these models are indistinguishable with respect to fit. Choosing between the zero-inflated and hurdle modeling framework, assuming Poisson and NB models are inadequate because of excess zeros, should generally be based on the study design and purpose [1]. If the study’s purpose is inference then modeling framework should be considered. For example, if the study design leads to count endpoints with both structural and sample zeros then generally the zero-inflated modeling framework is more appropriate, while in contrast, if the endpoint of interest, by design, only exhibits sample zeros (e.g., at-risk participants) then the hurdle model framework is generally preferred. Conversely, if the study’s primary purpose it is to develop a prediction model then both the zero-inflated and hurdle modeling frameworks should be adequate.

Cameron and Trivedi [13] report that ZINB and NBLH are a reparameterization of each other for a binary predictor. Again, in practice, there is no or little difference in AIC between the NB hurdle model and zero-inflated NB model [1]. However, it should be noted that NBLH which allows for over-dispersion and also accommodates presence of excess zeros, is more appropriate among all zero-adjusted models [9].

In the current paper, we only considered a single hurdle on infection and non infection for the study subjects for the hurdle negative binomial model. It should be noted that these can be extended to multiple hurdles [30] for the inclusion of measurement (i.e. *kato-katz*, *quantitative filtration technique* or other measures) error for instance, among other hurdles. The hurdle models can also be extended to capture spatial heterogeneity by introducing covariates and random effects [31]. We are currently working on multiple hurdles extension models.

In general, common factors to influence *S. haematobium* infection intensity and prevalence in both Malawi and Zambia studies. We found that *S. haematobium* infection intensity reduced with age, this confirms what previous studies found. In common intestinal helminths such as *Ascaris lumbricoides* (large roundworms) and *Trichuris trichiura* (whipworm) and also Schistosomiasis, children are more heavily affected and infected than adults [32]. Several other studies have reported that school-aged children show high infection intensity and prevalence [33–35]. Fishing in Shire river and working in gardens along the river were potential risk factors for exposure to Schistosomes and subsequent infection because transmission requires contact with the aquatic habitat of intermediate host snails [36]. This is in line with results from a study that was conducted in western Africa [37], that contact with water bodies that are a habitat for intermediate host snails is one of the main risk factors. Results showed low probability of infection for males compared to females. This could be explained by a number of factors including that Malawi being an agriculture based economy, and that mainly agricultural activities are carried out by females, hence they are more exposed to risk factors such as working in gardens and farming. Schistosomiasis is water dependent disease and the incidence is usually more amongst people who constantly get into contact with the schistosome infected waters through activities such as farming, fishing, swimming and washing [36].

Results showed that individuals who had received chemotherapy cure for helminth showed reduced risk of infection as well as infection intensity as compared to those in the control area in the Malawi study. Studies have shown that MDA significantly reduces Schistosomiasis infection [38, 39]. Evidence has shown that, following chemotherapeutic cure of *S. haematobium* infection, older individuals display a resistance to re-infection in comparison to younger children [40]. Therefore there is need to channel integrated control and interventions for helminths to areas with diseases burden in order to reduce and/or eradicate the infections - more especially towards school age children. Studies have shown that having one infection, is a risk factor for having other infections [41]. It is conceivable that the first parasite that establishes an infection may modulate the immune response in such a way that it makes it easier for the next [25].

We believe that the apparent dominance of agricultural, socioeconomic and demographic factors in determining *S. haematobium* infection risk in the villages carries important implications for disease surveillance and control strategies. Prevalence of *S. haematobium* was associated with age of an individual as well as working in the garden and also number of parasites an individual hosted. Furthermore, *S. haematobium* infection intensity was associated with gender, education level, garden, occupation and village type (intervention). Cercariae control control through environmental modifications and strategies involving socio-economic status improvement and MDA may be more promising approaches to disease control in this setting.

## Conclusion

The research reported in this article showed that the zero modified negative binomial logit hurdle (NBLH) and zero inflated negative binomial (ZINB) models are more appropriate methods for the analysis of data with an excess of zeros. These methods provide an alternative for analysing count data with more zeros than expected and eliminates the burden of data transformation to allow traditional methods of count data work. There are an increasing number of examples in the published literature where these "two-part" methods are being used for ZI data for helminths disease control planning and implementation programmes.

Ease of implementation and straightforward interpretation of the components and its direct link with the observed data used here, make the NBLH model particularly a valuable alternative for researchers analysing zero-inflated count data. It represents a key advance in the analysis of helminth disease data inflated with zeros.
